# Regulation as key to fulfilling the promises of agricultural genomics: Going beyond bottlenecks in plant gene technology development

**DOI:** 10.1111/tpj.70277

**Published:** 2025-06-16

**Authors:** Michail Ivanov, Emily A. Buddle, Rachel A. Ankeny

**Affiliations:** ^1^ School of Humanities University of Adelaide Adelaide South Australia 5005 Australia; ^2^ Philosophy Group Wageningen University Wageningen 6700 HB The Netherlands

**Keywords:** regulation, gene technology, biotechnology, agricultural genomics, genetic modification, gene editing, bottlenecks, public engagement in science, responsible research and innovation

## Abstract

The development of new gene technologies including gene editing has reinvigorated long‐standing global debates about if and how such technologies should be regulated. Many scientists working in agricultural genomics believe that current regulatory approaches are problematic, often emphasizing that the regulatory system is merely a ‘bottleneck’ that limits research and innovation in crop sciences. The concept of a ‘bottleneck’ is prominent in discussions in this domain, but we contend that what counts as a ‘bottleneck’ depends on point of view and the interests and goals of the party that wishes to describe a particular situation as bottlenecked. In this Focused Review, we provide a short account of recent scholarship on gene editing regulation and argue that regulation is an important part of the research development and innovation process that should not merely be viewed as a ‘bottleneck.’ Regulation permits regulators and diverse publics to engage with research and assess whether the particular application of gene technology is desirable and beneficial beyond the laboratory bench or field. We conclude by providing lessons for scientists working in agricultural genomics, emphasizing the need to move away from visions of ‘bottlenecks’ and embracing regulation's potential to support the promises associated with agricultural genomics.

## Significance Statement

This article provides a short review of recent scholarship on gene editing regulation and argues that regulation is an important part of the research development and innovation process. Processes associated with devising and enacting regulations permit regulators and diverse publics to engage with agricultural genomics research and its applications: scientists working agricultural genomics should stop considering regulation as a ‘bottleneck’ and embrace the potential for it to help fulfill the promises associated with agricultural genomics.

## INTRODUCTION

Research on the use of gene technologies in agricultural crops has been conducted for decades. Changes in methods, particularly through the recent development of gene editing (GE), have reinvigorated debates regarding whether and how gene technologies should be regulated. GE has allowed for more precise genetic changes than those produced with other genetic modification (GM) techniques (Ludlow, 2021). Plant scientists working in this field often represent the effects of GE as comparable to naturally occurring mutations in living organisms which do not involve the use of transgenes. For these reasons, many scientists working in agricultural genomics advocate for a more relaxed regulatory framework (or against the need for any regulation whatsoever) for GE (Ishii & Araki, 2017; Ludlow, 2021). There may be some scientists in this field who appreciate the structures and processes associated with regulation of biotechnology; limited evidence suggests more generally that there are divergent views among those in the scientific community about GM and GE (e.g. Bray & Ankeny, 2017) and its regulation (Selfa et al., 2021), a topic that warrants further investigation. In our experience working in this domain and based on attitudes documented in existing scholarly literature, many scientists working in agricultural genomics describe current governance approaches as problematic, often emphasizing that the regulatory system is a ‘bottleneck’ that limits research and innovation, including GE's potential global impacts (for explicit use of this terminology: Kumar et al., 2021; Mercé et al., 2020; Scheben & Edwards, 2018).

Since the concept of a ‘bottleneck’ has become prominent in discussions of regulation associated with GM and related biotechnologies, we use it as a springboard for the Focused Review presented in this article. The term is a metaphor that comes from the idea that the neck of a bottle limits the flow speed of the liquid contained in it. The concept is likely to be familiar to most in the context of traffic engineering, where bottlenecks are seen as largely structural and higher‐level problems, to be solved by reconfiguring road interchanges, changing traffic light patterns, and so on. It is more unusual at least in common parlance to view traffic bottlenecks as involving larger social drivers, such as the timing of when people travel to work or indeed whether they work at an office or from home. Talk of bottlenecks assumes that there would (and should) be flow through if the ‘bottleneck’ was not present, and thus that the goal is clear and agreed to by all (say, to get from point A to point B, in the case of traffic planning).

But what counts as a ‘bottleneck’ depends on one's point of view and the interests and goals of the party that wants to describe a particular situation as bottlenecked. For instance, in engineering and technological contexts, a bottleneck occurs where the performance or capacity of an entire system is severely limited by a single component (e.g. Santosuosso & Pinotti, 2020). In the context of plant transformation technologies, recent developments in GE have been heralded as clearing the long‐standing bottleneck related to the rapidly growing quantity of genetic data due to lower and more efficient high‐throughput technologies (e.g. Atkins & Voytas, 2020). These data could not be efficiently harnessed for gene discovery using traditional GM approaches given their relative imprecision among other technological issues, which in turn slowed down targeted identification and prioritization of agronomically important genes.

In regulation, bottlenecks are generally assumed to occur when inputs come at greater rates than the next step can convert them into outputs in the form of actionable and efficient regulation. Inputs are generated by various parties ranging from government itself, society, or scientists, among others, and in turn result in demands for more, less, or different regulations. Additional inputs occur in particularly in the context of rapidly emerging technologies, including the demands of scientists, governments, NGOs, and industry about the (supposed) urgency of the problems to be solved (on the problematic moral nature of ‘urgency’ claims in policy discourse, see Olson, 2015). In recent years in the context of plant transformation and agricultural genomics in particular, the problems to be solved have become explicitly aligned with broader, so‐called wicked problems including climate change, sustainability, and food security, as well as with global sustainable development goals and fostering a new ‘Green Revolution’ (Bain et al., 2020). In discussions of trade, lack of harmonization in global gene technology regulations has been described as creating a bottleneck, resulting in uncertainties and thus reduced investment in products made using gene technology (Mbaya et al., 2022; see also Eriksson et al., 2019) and barriers to innovation (Miller & Bradford, 2010, pp. 1013–1014; Smyth, 2020; Smyth et al., 2020)—a sentiment not confined only to discourse in peer‐reviewed publications (e.g. see Entine, 2018; van der Hoeven, 2011; similarly, see Banks, 2024).

In this Focused Review, we provide a concise and selective overview of recent social science scholarship associated with current gene technology regulation to generate insights particularly for scientists working in agricultural genomics about this view of regulation as a ‘bottleneck’ and discuss the role of regulation, its relation to social values, and how reframing conversations about regulation could result in more fruitful discussions about the future of agricultural genomics and plant biotechnologies. We contend that regulation is an important part of the innovation process, introducing ways for different publics to engage with research to understand if the technological application is considered to be desirable and beneficial beyond the laboratory bench or field trial. We present guidance for natural scientists about how to better engage with regulatory processes, and outline a series of open questions which require additional, robust empirical research in order to encourage science studies and policy scholars to continue to investigate this complex, rapidly moving, and important domain.

## WHAT IS REGULATION?

Scientific literature related to gene technology often fails to define what is meant by the term ‘regulation.’ When this article discusses ‘regulation,’ it adopts a definition which views the state (i.e. a geographic locale considered to be organized as a political community governed by a specific government) as the central controlling entity (for purposes of this article, our account draws heavily on Black, 2002). Although we recognize that many issues, including gene technology, are regulated in part under international frameworks (e.g. through the Cartagena Protocol on Biosafety), this article focuses on the individual state. This approach is adopted because it is often the state's government, as underpinned by a given society's values, which decides whether to adopt, or reject, a particular international regulatory instrument (Ishii & Araki, 2016). International organizations and instruments also play roles which are essential to the broader regulatory environment; however, this article focuses on the state because it is typically the primary regulator and hence also frequently associated with claims about regulatory bottlenecks. When it comes to the regulation of GE, there is limited global consistency, with different states taking different approaches and using distinct processes (Ishii & Araki, 2016). ‘Regulation’ describes the instruments created by a state's government (whether legal, quasi‐legal, or non‐legal) which impose obligations or constraints on actors and which may be enforced and accompanied by sanctions for non‐compliance. These instruments include acts of parliament, laws, delegated legislation, policies, orders, licenses and associated requirements, codes, and guidelines.

To adapt Foucault's terminology with regard to governance, regulation is what structures the ‘conduct of conduct’ (Foucault, 1991). It is assumed that various actors will continue to behave in various ways that are constantly evolving in the absence of intervention via regulation (what is sometimes misleading called ‘autonomy’ or ‘freedom to operate’ by scientists, as we discuss in more detail below), and thus regulation seeks to produce changes in these actors' behaviors. Therefore, regulation can be viewed as a system built out of interdependent parts (such as the instruments described above) which are embedded into a legal system, which in turn is itself a part of the broader social system (Sheehy & Feaver, 2015) on which it is designed to have effects, particularly to shape agents' behaviors.

The specific forms that regulatory systems take are influenced by the anticipated attitudes of the regulated agents, particularly toward compliance. For instance, self‐regulation is often utilized when it is assumed that agents can be trusted to comply with certain norms or requirements largely without formal enforcement mechanisms. Where the legal system is in tension with the social system including public expectations, some social actors are likely to oppose regulations associated with the domain in question (Sheehy & Feaver, 2015). Hence public engagement is a central tenet of regulatory processes and should be viewed as a mutual learning process rather than mere ‘window dressing’ (Svingen, 2022; see also Svingen & Jahren, 2024). No single actor (or group or type of actors) has the expertise and capacity to shape regulation in isolation, and outcomes can be severely restricted both by the limits of knowledge but also lack of coordination with other parties (Black, 2002). Regulation is often politicized and ideologically based, with actors advancing arguments (based on evidence or otherwise) that encourage publics to accept (or reject) particular regulations (Lassoued et al., 2021; Sheehy & Feaver, 2015).

The ideological bases of regulation are prominently shown in popular discourse when it comes to court‐made regulations, such as the precedents established by the Supreme Court of the United States (Weinshall et al., 2018) or the European Court of Justice's 2018 ruling on effectively regulating GE as GM (Gelinsky & Hilbeck, 2018). Certain regulatory spaces, such as those related to economics or the sciences, are represented as being motivated by scientific and objective evidence, but may instead be laden with normative ideas (Sheehy & Feaver, 2015). Similarly, regulation can, both advertently and inadvertently, restrict certain groups' previously enjoyed freedoms (see, e.g. Kailis, 2013, relating to the loss of public fishing rights, including the traditional fishing practices of Indigenous Australians by virtue of fisheries management legislation). Extensive empirical research in the social sciences shows that publics and governments do not experience technological advances in biotechnology in isolation, and instead document how public perceptions of technology influence public acceptance, regulations, and governance of technology, including those used in agricultural genomics, including GE (Macnaghten & Habets, 2020; Middelveld et al., 2022; Montenegro de Wit, 2022; Nawaz & Satterfield, 2022). Failing to recognize societal and ethical concerns present in any regulatory framework is likely to cause persistent tensions, even where those concerns may not appear to be directly related to the regulations. Such concerns can nevertheless be addressed or alleviated through regulation not only by allowing a certain technology or imposing a moratorium or restrictions on its use, but also by committing to more openness, recognizing underlying values and assumptions, considering alternatives, and moving beyond simplified decisions to more nuanced consideration of how, under what conditions, and whether and when technologies should move ahead (Hartley et al., 2016).

By virtue of its state‐centricity, particular regulations often are not harmonized with those of other states, including at a global level, which is seen as a significant problem by many scientists (Ramos et al., 2023). Lack of harmonization can result in practical differences between states in the ways in which actors can conduct themselves. Regulatory approaches of gene technologies vary within and across jurisdictions, shaped by the interrelationships between governance, publics' perceptions, and the potential impacts of the technology within the specific context in question. There also are a variety of ways in which regulation of gene technology occurs, ranging from introducing new, specific legislation to modifying existing frameworks to accommodate technological advances such as GE (Eriksson et al., 2019; Friedrichs et al., 2019; Tachikara & Matsuo, 2023). Lack of harmonization in legislation can lead to conflicts or misunderstandings, for example relating to different labelling requirements across states (Svingen & Jahren, 2024) or the creation of GE‐free zones for growing food in close proximity to agricultural areas without these restrictions. However, variation in publics' perceptions in each locale means that harmonization can never be absolute, but a balance can be struck by involving a range of different interested intra‐ and inter‐state actors (Ramos et al., 2023; Svingen, 2022).

Hence although regulation of gene technology might be perceived by some as creating ‘bottlenecks,’ regulatory frameworks for the effective use of a new technology such as GE can be structured to align developments and applications with societal expectations (Lassoued et al., 2021; Svingen, 2022). If application of a technology is shown to be successful in achieving its aims, typically including being beneficial and not injurious to society, regulation can be developed so as to further the potential of the technology while still maintaining public trust (Kuzma et al., 2023; Svingen, 2022). In this way, rather than being a bottleneck, appropriate structured regulation can act as a step in the broader innovation and research development process and as an opportunity for all interested actors to consider potential impacts of a certain technology and the definition of limits. Thus, regulation can be conceptualized as a filter through which the metaphorical liquid (the biotechnology in question, in our case GE) passes before it can come out of the bottle. It acts to remove many elements that could cause tensions or harms of various kinds to society or publics within it. Where regulation does not meet key societal goals, its impacts can be just as harmful as under‐ or over‐regulating (Sheehy & Feaver, 2015).

## REVIEW OF RECENT SCHOLARSHIP ON VIEWS ON TECHNOLOGY REGULATION

There is copious literature documenting the complexities associated with gene technology regulation and perceptions of it in different locations; however, space limitations prevent us from providing a complete survey of even the recent literature on the regulations themselves (see e.g. Ishii & Araki, 2017; Turnbull et al., 2021), which are rapidly evolving. Instead, we focus selectively on scholarship in this domain that specifically focuses on regulation in relation to societal expectations and demands, with emphasis on agriculture‐related gene technologies.

Scientists' views dominate much of the literature that practicing plant scientists are likely to encounter: the narrative here tends to be that GM has been stymied by technological limitations and regulation, and GE could and should be seen as a distinct technology that can overcome the technological bottlenecks in the field; however, it is emphasized that there are likely to continue to be (implicitly inappropriate and undesirable) regulatory bottlenecks. The potential of GE, these critics of regulation argue, is being undermined in a number of states by process—rather than product‐based regulation, which inaccurately overlooks the similarity between the effects of GE techniques and those of naturally occurring mutations (Ludlow, 2021; Scheben & Edwards, 2018). Thus, the argument continues, despite the availability of techniques which are more accurate and cost‐ and time‐efficient, regulatory frameworks prevent their use, which in turn hinders critical and important global goals, such as the closure of the food gap by the target year of 2050 (Lassoued et al., 2021). In contrast, the scholarly theoretical and empirical literature on regulation in this domain by social scientists provides a more nuanced account of recent and current developments: we summarize some of the main themes below.

Studies that focus on public attitudes to biotechnology can typically be divided into two types of approaches: those that explore consumers' views and those that engage with broader communities or publics, due to the different ways in which consumer versus citizen behaviors play out in these domains (Brom, 2000). Behaviors and views related to purchasing and consuming food tend to be studied using methods from consumer studies (including marketing, economics, and so on), while views on policy, regulation, or other broader social topics are explored using social science methodologies with people viewed as citizens who vote (directly on policy or indirectly for representatives who make policy and legal decisions) or community members who can engage in decision‐making with the greater good in view (Ankeny, 2016, 2018; Grant et al., 2021; Wilkins, 2005). Most individuals are both consumers and citizens, depending on the specific focal topic, but the behaviors associated with each category are often distinct, performed at different points in time and in different manners. Most importantly, although one may not consume a particular product (e.g. GM food or, say, meat or animal products), their views on what regulations should be in place at the societal level in association with these products may not be directly aligned with their individual food choices (e.g. Ankeny & Bray, 2016; Bray & Ankeny, 2017; Phillipov et al., 2024).

Recent research has shown complexities associated with public attitudes toward GE as compared to GM techniques, and particularly between cis‐ and transgenic approaches, which has flow‐on effects with regard to views on regulation (Kuzma, 2023). For example, cisgenic modifications are often considered by many publics to be more ‘natural’ and thus more acceptable than transgenic modifications (Mielby et al., 2013; Myskja, 2006; Rommens et al., 2007). Similarly, although researchers found a higher level of acceptance of cisgenic GE in contrast to transgenic GM technologies in the UK and Switzerland, public acceptance also was dependent on whether the application was perceived to be beneficial (Bearth et al., 2022; see also Ipsos Mori, 2021). A recent study of US consumers documented that people were more willing to consume foods created using CRISPR as opposed to first‐generation GM or transgenic foods, but both were viewed less positively than conventional foods (Shew et al., 2018). Furthermore, a survey in the United States showed that providing information about the potential health and environmental benefits of GM and GE did not affect consumers' willingness to consume foods made with these technologies but prior perceptions about agricultural biotechnology, safety and benefits, and who developed the technology were the most important factors in relation to willingness to consume (Paudel et al., 2023).

Other studies have found that community members are generally less interested in the precision of GE techniques or from where the genes originate (in transgenic applications) and instead tend to focus on whether these applications form part of industrialized practices that are seen as now dominating agricultural production, leading them to ask whether there are alternative approaches in locales including Australia, Canada, and the United States (Ankeny et al., 2022; Nawaz et al., 2023; Nawaz & Satterfield, 2022). Previous research has emphasized that the social and cultural contexts within which agricultural gene technologies are being applied matter to publics' views on it. For example, Busch et al. (2022) conducted an online survey in Canada, the United States, Austria, Germany, and Italy and recorded significant diversity in views. Hence, they conclude that debates and discussions about biotechnology regulation, including GE, should not simply consider whether it should be permissible to use this technology but should delve more deeply into which applications may be appropriate for different purposes and in different locales. This finding supports the view of regulation as a filter rather than a bottleneck in that it allows for the removal of inappropriate elements (whether in the approach or substance) of a certain biotechnology before it is approved for use in alignment with its obligations to society.

Some studies have focused on scientific or subject matter expert (SME) perspectives on the regulation of gene editing. Much of this research finds that scientists and other types of experts believe that emerging biotechnologies hold great promise for addressing social and climate change‐related challenges, but often view regulation of gene technologies as ambiguous or difficult to navigate, which they contend limits the ability for market growth (Lassoued et al., 2021; Ruder & Kandlikar, 2023). A US study found that SMEs believe that GE should be subject to less regulation due to the technological differences between GM and GE, and that GE makes the engineering process so trivial that the slow nature of current risk‐based regulatory approaches may not be fit for purpose, although biotechnology developers did note the need for greater public engagement in GE governance (Kuzma et al., 2016). In contrast, Ruder and Kandlikar (2023) found that Canadians continued to view public engagement and risk mitigation with regard to gene technologies as the responsibility of government. van der Berg et al. (2021) highlight publics' appetites for future‐proofed legislation with regard to genomic food technologies among their Dutch participants, echoing other authors' claims about the need for nuance in regulatory decision‐making (e.g. Hartley et al., 2016).

Part of the issue with the attitudes of scientists working in agricultural genomics toward regulation and governance appears to be that they continue to see their work as ‘value‐free,’ separate from society, and as related to producing knowledge that leads to unproblematic societal benefits through industry. In a qualitative study of Dutch university‐based scientists, respondents rarely considered the GE crop debate, or their own positions in it, to be value‐based, and struggled to recognize the validity of viewpoints other than their own, with slightly better results among those who had collaborations with social scientists (So et al., 2024).

For instance, Mercé et al. (2020) describe the gap between ‘scientific knowledge regarding the impact of new plant breeding techniques on human and environmental health’ and ‘the current legislation [across the world]’ as a ‘major bottleneck’ to the expansion of GE crops. This type of claim appears to exclude consideration of the values underpinning both the scientists' work and any legislation, instead focusing solely on the benefits that this technology can provide. The authors consider much legislation to be ‘obsolete’ because they fail to distinguish between GM and GE technologies, which is reasonable in a purely technical sense, but does not appear consider that attention must be given to more than just the ‘scientific and economic’ impacts of any technology. The role of filter is thus appropriate and indeed required in regulatory processes which seek to be in alignment with social norms while nonetheless supporting new developments, and ought not to be seen as a hinderance or bottleneck. In comparison, a U.S. interview study of representatives of regulatory agencies, commodity groups, consumer and environmental NGOs, biotechnology and food industries, and scientists found new and unexpected alignments between many of these actors in response to demands for broader engagement of publics and greater transparency in the governance of biotechnologies in agriculture and food. Shared desires to incorporate lessons from the debates over GM labelling shaped their responses which included calls for new forms of regulatory oversight, particularly among non‐state actors including industry, rather than reliance on self‐regulation (Selfa et al., 2021).

## GUIDANCE FOR SCIENTISTS ON ENGAGING WITH AND EMBRACING REGULATION

The empirical research highlighted above documents that early and frequent engagement with publics can allow deeper understanding of their potential concerns. There are several approaches for such engagement: for instance, interdisciplinary research projects related to agricultural genomics that include scholars with social science methodological expertise can help to support greater engagement with sociocultural understandings of and attitudes toward gene technology development and implications for regulation. It is clear that the type of application of gene technology matters more than whether it is GE or GM. Some applications are more likely to be accepted than others and, in turn, to support less restrictive regulatory regimes. For instance, in an Australian study of attitudes to gene editing in red meat production, applications of gene editing that had perceived benefits for animal welfare were generally viewed more favorably than those considered to be merely cosmetic (e.g. changing coat color) (Ankeny et al., 2022; see also Busch et al., 2022; Middelveld et al., 2022).

Some scientists have claimed that the potential economic benefits of certain gene technologies are significant enough to justify the removal of regulatory limitations or perhaps all regulation whatsoever, particularly given similarities between GE and traditional breeding techniques. However, as noted above, economic drivers are not the only motivations that should be considered in regulatory processes, and the default position should not be seen as a neoliberal, laissez‐faire approach particularly with complex issues tied to a range of societal concerns. It is important to note that some regulatory regimes explicitly exclude consideration of economic, ethical, and/or social benefit in technology assessment practices, for instance the long‐standing Commonwealth *Gene Technology Act* in Australia (see Rogers, 2002).

Who is likely to benefit from the use of gene editing technologies, and in what ways, deeply matters to a range of publics. There is documented increasing distrust of industrial players, particularly in the domain of food and agriculture (consider the pejorative terminology of ‘big food’), due to perceptions that the main goal of these players is to generate what are considered to be excessive profits, which is particularly problematic for many given that food is viewed as a right (Weale, 2010; Yates et al., 2021). Agricultural genomics research associated with industry or potential applications of gene technologies to the food supply must contend with these social and cultural drivers when considering the ways in which regulation in this domain is created and implemented.

Further, it cannot be assumed that scientific or biological ‘equivalency’ implies social ‘equivalency,’ and in turn social acceptance. Many scientists have argued that certain forms of GE should not be regulated (and should become preferred approaches for crop breeding) because the resultant plants will be transgene‐free and are likely to fall outside of standard regulatory definitions of GM in various locales, and hence, this will lead to greater social acceptance of these crops. Such claims tend to be grounded in the idea that such plants would not be distinguishable from those grown using traditional or conventional breeding methods such as random chemical metagenesis, and hence rely on a product‐oriented (rather than a process‐focused) definition of what counts as GM. They also imply particular visions of the likely enforcement mechanisms associated with any regulations as necessarily grounded in screening of the final products, rather than, say, on audit of processes. However, some commentators have claimed that these types of crops also might fall outside some forms of process‐focused regulations (Camacho et al., 2014), while others have encouraged abandoning the framing of the debate around ‘product versus process’ altogether (Kuzma, 2016; Sprink et al., 2016). The debates continue, including in relation to implications for intellectual property and patenting, trade including export and import, and labelling. These considerations go well beyond assertions of academic freedom, the autonomy of science, or ‘freedom to operate’. The standard definition of freedom to operate refers to being able to use a product or process without infringing on intellectual property rights. However, scientists often invoke the notion of ‘freedom to operate’ and its supposed precarious status when discussing the requirements imposed by regulation on their research and its applications, and particularly supposed ‘bottlenecks,’ including in agricultural genomics.

Regulation, which this article has argued should be conceptualized as more of a filter than a bottleneck, allows for changes or tweaks to an innovation so as to make its use more responsible. It serves as part of a broader social system on which it is designed to have effects, particularly on shaping agents' behaviors, to ensure they are aligned with societal expectations. Thus regulation is not all about the economics or the science: as noted by social scientist Kuzma et al. (2016) based on her research and policymaking experiences related to biotechnology, ‘it is impossible to be completely ‘science based’ in a regulatory system’ (p. 167). Regulatory decisions are always about value judgments particularly when safety, risk, and benefits are at issue. Hence the kind of empirical evidence that science provides is only one part of the picture: we also need robust evidence about views and concerns among interested publics, and how regulation should be structured in light of these.

As science policy expert Sarewitz (2015) has stressed, the idea that the risks, benefits, and ethical challenges of emerging technologies are something to be decided by experts is ‘wrong‐headed, futile and self‐defeating. It misunderstands the role of science in public discussions about technological risk. It seriously underestimates the democratic sources of science's vitality and the capacities of democratic deliberation. And it will further delegitimize and politicize science in modern societies’ (p. 413). Hence, decisions about regulation of GE have considerable broader implications particularly in today's so‐called ‘post‐truth’ environment.

There is growing attention to social and ethical implications of biotechnologies within regulatory processes. In Norway, for instance, the *Gene Technology Act* of 1993 requires that every GMO risk assessment must, in addition to its safety, consider the social utility and ethics of said GMO (Myskja & Myhr, 2020). This assessment is done with the goal of using GMOs in an ‘ethically justifiable and socially acceptable manner’ (Myskja & Myhr, 2020). Over 40 states have similar regulations that require consideration of socio‐economic impacts (in which economic impacts do tend to prevail over the social) in their domestic GM crop regulation (Catacora‐Vargas et al., 2018). Even the Cartagena Protocol on Biosafety recognizes that parties to the Protocol *may* take into account ‘socio‐economic considerations arising from the impact of living modified organisms on the conservation and sustainable use of biological diversity’ (Article 26; see Myskja & Myhr, 2020). Still, as Catacora‐Vargas et al. (2018) found, there is a ‘neglect of attention to social aspects of GM crops’ in research, and this absence is clear in the evidence typically relied on in policymaking.

Everyone has interests, even scientists: hence exclusion of various voices due to their social, political, economic, or other interests, or perceptions about their (lack of) knowledge and expertise (associated with the discredited ‘deficit model’: for a recent discussion, see Grant, 2023) is neither morally or pragmatically useful (see also So et al., 2024). Rather than viewing certain types of groups (such as NGOs) as merely obstructionist and ideologically motivated, it is critical to develop a richer understanding of their positions in the broader contexts in which they are being asserted, which often are not simply for or against plant gene technology. For instance, empirical research comparing the positions of NGOs and the U.K. Nuffield Council on Bioethics (a leading policy authority in the United Kingdom, generally viewed as a reasonable and independent think tank) found that although NGOs aimed to broaden the scope of debate to include deeply political questions regarding agricultural and technological choice, these groups shared a significant number of concerns about the social and ethical implications of GE with regard to problem or solution framing, definitions and terminology, implications of regulatory and research choices on consumer choice, and power relations (Helliwell et al., 2019). Raising questions and fostering discussion are signs of a healthy society which has robust regulatory and policy processes.

## CONCLUSIONS

There is no one simple set of recommendations for scientists working with agricultural genomics and other emerging biotechnologies about how to engage (and even embrace) regulation, given the diversity of applications, publics, and societies facing these issues. This paper has aimed to generate reflections for consideration about the role of regulation and its relation to social values and goals, in order to promote dialog particularly among scientists working in agricultural genomics about reframing conversations on regulation. Rather than viewing regulation merely as a ‘bottleneck,’ seeing it instead as a useful filter could result in more fruitful discussions about the future of agricultural genomics and plant biotechnologies. There is rich empirical social science literature that addresses many important issues in this domain, but there are also open questions that require deeper and ongoing reflection (whether through research or in practice) by scientists using agricultural biotechnologies as these technologies continue to develop, most fruitfully in collaboration with social scientists and regulators themselves (see Box 1).

Box 1 **Questions for scientists working with agricultural gene technologies to consider**

1. Due to the speed at which GE technologies are changing agricultural crop breeding practices and increasing perceptions about the urgency of the issues that can be addressed using them (such as climate change and food security), are there more innovative and agile regulatory approaches that should be proposed?
2. How should scientists‐in‐training be supported to build skills about assessing and understanding the broader implications of their research, including with regard to regulation and engagement of publics?
3. At what stage or level (if any) and in what ways should scientists working in agricultural genomics be engaged about the development and applications of regulation of biotechnology?
4. What approaches to fostering greater engagement among scientists working in agricultural genomics with publics and regulators are likely to be most productive, particularly where there have been long‐standing conflicts, for instance, with NGOs?
5. Are there ways in which policymakers, farmers, and others in the agricultural sector, and other key players involved in the regulation of agricultural genomics can be engaged and involved early in technology development processes, and what effects might such engagement have on regulatory processes?

We contend that robust regulatory processes are a critical part of any responsible research and innovation process, as they require that different publics and viewpoints be considered to assess whether a technological application is likely to be socially desirable and beneficial beyond the laboratory or a field trial, among many other considerations. Indeed, a failure to properly consider such viewpoints may itself lead to a ‘bottleneck’ or impediment to innovation, as others' viewpoints (when unvoiced or unheard) may well become more oppositional and entrenched. All types of scientists working in agricultural genomics should collaborate both with policymakers and publics on regulation of agricultural gene technologies, and also with social science scholars who have expertise in science, regulation, and policy to continue to investigate and come to deeper and more positive understandings of the complex, rapidly moving, and important role of regulation in this domain.

## CONFLICT OF INTEREST STATEMENT

All authors are affiliated with the ARC Training Centre for Future Crops Development (IC210100047) (https://futurecropscentre.edu.au) as part of Program 2: ‘Develop responsible research innovation practices’ and receive research funding from it. EAB and MI receive partial salary support from the Training Centre. RAA is a member of the Gene Technology Ethics and Community Consultative Committee (GTECCC) which is an advisory committee associated with the Australian Commonwealth Gene Technology Regulator (OGTR) and of the Social Science and Economics Advisory Group (SSEAG) for Food Standards Australia New Zealand (FSANZ). She is a recent past member of the GM Crop Advisory Committee, Government of South Australia, and EAB is a current member.

## Data Availability

Data sharing not applicable to this article as no datasets were generated or analysed during the current study.
